# Maternal Gestational Smoking, Diabetes, Alcohol Drinking, Pre-Pregnancy Obesity and the Risk of Cryptorchidism: A Systematic Review and Meta-Analysis of Observational Studies

**DOI:** 10.1371/journal.pone.0119006

**Published:** 2015-03-23

**Authors:** Lin Zhang, Xing-Huan Wang, Xin-Min Zheng, Tong-Zu Liu, Wei-Bin Zhang, Hang Zheng, Mi-Feng Chen

**Affiliations:** 1 Department of Urology, Zhongnan Hospital of Wuhan University, Wuhan, Hubei, People’s Republic of China; 2 Department of Obstetrical, Jingmen No. 2 People’s Hospital, Jingmen Hubei, People’s Republic of China; Harvard Medical School, UNITED STATES

## Abstract

**Background:**

Maternal gestational smoking, diabetes, alcohol drinking, and pre-pregnancy obesity are thought to increase the risk of cryptorchidism in newborn males, but the evidence is inconsistent.

**Method:**

We conducted a systematic review and meta-analysis of studies on the association between maternal gestational smoking, diabetes, alcohol drinking, and pre-pregnancy obesity and the risk of cryptorchidism. Articles were retrieved by searching PubMed and ScienceDirect, and the meta-analysis was conducted using Stata/SE 12.0 software. Sensitivity analysis was used to evaluate the influence of confounding variables.

**Results:**

We selected 32 articles, including 12 case—control, five nested case—control, and 15 cohort studies. The meta-analysis showed that maternal smoking (OR = 1.17, 95% CI: 1.11–1.23) or diabetes (OR = 1.21, 95%CI: 1.00–1.46) during pregnancy were associated with increased risk of cryptorchidism. Overall, the association between maternal alcohol drinking (OR = 0.97, 95% CI: 0.87–1.07), pre-pregnancy body mass index (OR = 1.02, 95% CI: 0.95–1.09) and risk of cryptorchidism were not statistically significant. Additional analysis showed reduced risk (OR = 0.89, 95% CI: 0.82–0.96) of cryptorchidism with moderate alcohol drinking during pregnancy. No dose—response relationship was observed for increments in body mass index in the risk of cryptorchidism. Sensitivity analysis revealed an unstable result for the association between maternal diabetes, alcohol drinking and cryptorchidism. Moderate heterogeneity was detected in studies of the effect of maternal alcohol drinking and diabetes. No publication bias was detected.

**Conclusion:**

Maternal gestational smoking, but not maternal pre-pregnancy overweight or obesity, was associated with increased cryptorchidism risk in the offspring. Moderate alcohol drinking may reduce the risk of cryptorchidism while gestational diabetes may be a risk factor, but further studies are needed to verify this.

## Introduction

Cryptorchidism is a genital malformation of newborn males, in which one or both testes are absent from the scrotum at birth. It has a prevalence of 1.63 to 2.90% [1, 2], and in approximately 70% of affected infants, the testes spontaneously descend during the first 3 to 6 months after their birth [3–5]. Nearly 20% of undescended testes are impalpable [6]. Cryptorchidism can be treated surgically or by hormone therapy, and may have severe long-term complications if untreated. Evidence presented in recent reviews indicates that cryptorchidism carries a high risk of infertility and testicular cancer [7, 8].

The causes of cryptorchidism are not well understood. In addition to genetic alterations [9] and exposure to endocrine-disrupting chemicals [10], maternal smoking [10–13], alcohol drinking [12–14], diabetes [15], and pre-pregnancy obesity [16] are considered potential risk factors for cryptorchidism. However, the mechanisms underlying these relationships remain unclear and the results of epidemiologic studies of associations between maternal exposure to specific risk factors and cryptorchidism are inconsistent. The relationship between maternal gestational smoking and risk of cryptorchidism was assessed in a recent review [17]. But there were some limitations in the methodology; and the studies it included were insufficient. Additional meta-analysis [18] has been well designed, but several studies have published after that. And none of these two meta-analyses have focused on the remaining risk factors mentioned above. The evidence available in recent reviews is thus not comprehensive.

We conducted a systematic review and meta-analysis of current observational studies following the PRISMA statement [19] (S1 PRISMA Checklist). Our primary aim was to quantitatively evaluate the association of maternal gestational smoking, diabetes, alcohol drinking, and pre-pregnancy obesity with the risk of cryptorchidism.

## Methods

### Eligibility criteria

The studies selected for analysis satisfied the following criteria. (1) The studies included newborn males diagnosed with cryptorchidism in case group. (2) The effects of maternal gestational smoking, diabetes, alcohol drinking or maternal pre-pregnancy body mass index (BMI) were investigated. (3) The control or non-case group comprised boys without cryptorchidism. (4) The study was of a cohort, case—control, or nested case—control design. (5) Complete data were reported; or missing data could be obtained from the study investigators. Response letters or meeting papers were also considered if additional information was provided.

### Search strategy

We searched PubMed and ScienceDirect for relevant, English-language studies published to February 10, 2014. The PubMed search terms were: [(gestational diabetes OR gestational glycuresis) OR (gestational smoking OR gestational cigarette exposure) OR (gestational alcohol drinking OR gestational drinking) OR (pre-pregnancy obesity OR pre-pregnancy overweight OR pre-pregnancy body mass index) OR (risk factors)] AND (cryptorchidism OR undescended testes OR cryptorchism) AND (human). We also manually searched the reference lists of retrieved articles and reviews.

### Data extraction

Two reviewers (L. Zhang and X.-H. Wang) independently extracted the author names, publication year, country, study type, the number of cryptorchidism cases, controls or non-cases or person years, exposure categories, adjusted or crude relative risk (RR), odds ratio (OR), hazard ratio (HR), or prevalence ratio (PR) with 95% confidence intervals (CIs), and adjusted variables. A standardized data collection form was used. When more than one OR (RR, HR, or PR) was shown, the one from multivariate-adjusted was extracted to account for the influence of potential confounders. If a study included two or more control groups, the one that matched more of the study variables or was population-based was analyzed. A third author (T.Z. Liu) independently reviewed the articles for the extracted information, which were then discussed by all authors to decide on inclusion.

### Statistical analysis

In the first part of the analysis, we pooled the ORs and 95% CIs of cryptorchidism for the mother who smoking, drinking, with gestational diabetes or pre-pregnancy obesity. For studies that did not provide valid evaluations, we used the reported tabular data to calculate crude ORs with their 95% confidence intervals (CIs). If the raw data included a zero cell, 0.5 was added to every cell in the 2 x 2 table. Studies with two or more zero cells in 2 x 2 tables were excluded. In the meta-analysis, hazard ratios (HRs) and prevalence ratio (PRs) were considered as RRs; and then relative risk (RRs) and relevant standard error (SE) were converted into ORs using the method described by Zhang et al [20], which requires the incidence of the outcome of interest (P_0_) in the control group. It should be noted that, this conversion may overestimate the variance of OR [21]. We pooled RR with OR directly if P_0_ could not be obtained since the incidence of cryptorchidism is low (< 10%) that RR is similar to OR, as described by Zhang et al. [20]. We used I^2^ statistics to detect statistical heterogeneity, which varied from 0 to 100% and was described as low (0–40%), moderate (30–60%), substantial (50–90%), and considerable (75–100%) [23]. A fixed-effect model was applied when slight heterogeneity (I^2^ < 30%) was detected; otherwise, a random-effect model was used. Studies that reported results separately by area, sex, or race, the ORs were pooled using a fixed-effects model before combining it to overall meta-analysis. Subgroup analysis (such as geographical region, study design) was used to address heterogeneity. The ORs and 95%CI were log-transformed and the weight was calculated by the inverse variance method.

To determine whether high and low exposure resulted in differences in the risk of cryptorchism (such as alcohol drinking), we pooled the ORs of high or moderate separately, and compared them to ORs of low exposure groups. We also evaluated potential dose-response relationships as previously described by Orsini et al. [24] when a sufficient number of studies reported relevant data for the 4 risk factors (such as BMI). Briefly, a regression model was initially fitted within each study to estimate the RR of cryptorchism for each unit of increase in exposure to a risk factor. Then the RRs in each individual study were pooled in a random-effect model. RR was considered as a general term that included RR, OR, HR, or PR [25]. The assigned doses were estimated as the midpoints of the upper and lower boundaries of each category [25]. For open-ended lower categories, the assigned dose was assumed to be 0.83 (1/1.2) times the cutoff point [26]; for open-ended upper categories, the assigned dose was evaluated as 1.2 times the cutoff point [25].

Egger’s test and Begg’s test were used to detect potential publication bias, and a sensitivity analysis was performed to evaluate the robustness of the results. All the analyses were conducted using Stata/SE 12.0 software (StataCorp LP, College Station, Texas, USA).

## Results

### Search results and study characteristics

**Fig 1:**
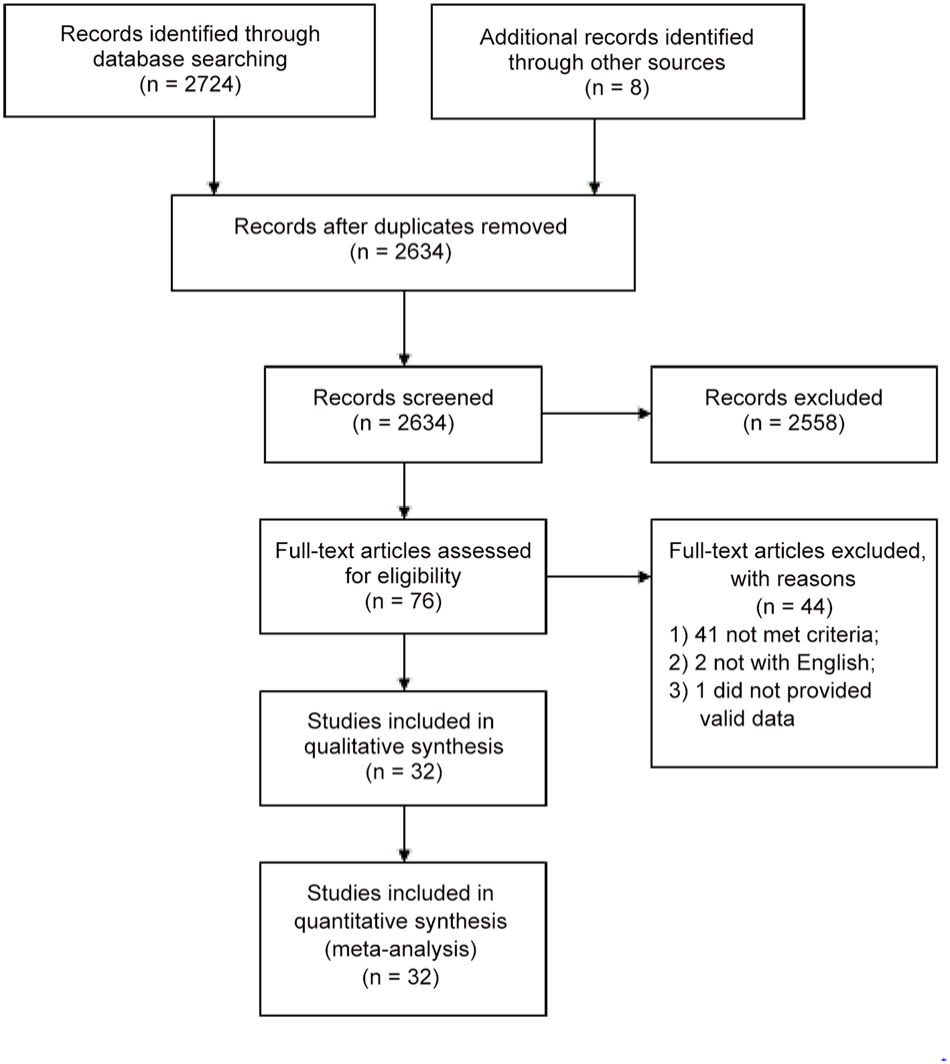
Flow diagram of the literature search.

Thirty-two studies were selected [2, 10, 12–16, 22, 27–50]. Among them, there were 12 case—control (all matched with age and sex), five nested case-control, and 15 cohort studies (at least 1 years follow-up), with a total number of 21,774 cryptorchidism cases. Fig. 1 shows the flow diagram. Ten studies were conducted in the United States, seven in Denmark, three in the UK, two in Japan, two in the Netherlands, two in Italy, and one each in Egypt, France, Spain, Sweden, and Lithuania. Two studies, [28, 37] were published by the same author in different years, and four [30, 40] and [34, 41] were studies published by two different authors in different journals. The study [49] was a response letter by Main [48] that reported the information of maternal alcohol consumption and risk of cryptorchism that was not presented in study [48]. Duplicate data were excluded when reported in two or more studies. The study characteristics were shown in Table 1, more complete data were given in S1 Dataset.

**Table 1:** Characteristics of included studies in the meta-analysis.

First Author	Year	Country	Study types and description	Total cases/controls	Risk Factors
Zakaria	2013	Egypt	Nested case-control study with 1 year follow-up.	29/40	Maternal smoking, diabetes.
Brouwers	2012	Netherlands	Hospital based case-control study matched with geographical distribution and sex.	200/629	Maternal smoking, alcohol, diabetes, BMI.
Jensen^1^	2007	Denmark	Cohort study with 3 years follow-up.	270 cases	Maternal smoking.
Strandberg	2009	Denmark	Cohort study with 6 years follow-up.	1,598 cases	Maternal alcohol.
Kurahashi	2005	Japan	Hospital-based case-control study matched with sex.	96/116	Maternal smoking, alcohol, BMI.
Adams	2011	America	Population-based case-control study matched with age and sex.	2,759/35,268	Gestational smoking, diabetes, obesity.
Berkowitz^1^	1996	America	Nested case-control study with 3 years follow-up.	69/219	Maternal smoking, alcohol, diabetes, BMI.
Mori	1992	Japan	Hospital-based case-control study matched with age and sex.	104/104	Maternal smoking, alcohol.
Agopian	2014	America	Cohort study with 10 years follow-up.	4,001 cases	Maternal diabetes.
Trabert	2014	America	Cohort study with 10 years follow-up.	3,649 cases	Maternal diabetes.
Mongraw-C	2007	America	Cohort study with 8 yeas follow-up.	101 cases	Maternal smoking, alcohol.
Damgaard^1^	2008	Denmark	Cohort study with 2 to 4 years follow-up.	128 cases	Maternal smoking, alcohol.
Fernandez	2007	Spain	Nested case-control study with about 2.5 years follow-up.	47/105	Maternal smoking, BMI.
McGlynn	2006	America	Cohort study with7 years follow-up.	238 cases	Maternal smoking, diabetes.
Jones	1998	British	Cohort study with 17 years follow-up.	1,499 cases	Maternal smoking, obesity, diabetes.
Giordano	2008	Italy	Population-based case-control study matched with age and sex and city.	48/202	Maternal smoking, alcohol.
Jensen^2^	2007	Denmark	Cohort study with 3 years follow-up.	270 cases	Maternal smoking, alcohol.
Virtanen	2006	Denmark, Finland	Hospital-based case-control study matched with age and sex.	125 cases	Maternal smoking, gestational diabetes.
Pierik	2004	Netherlands	Nested case-control study with 2 years follow-up.	78/313	Maternal smoking.
Biggs	2002	America	Hospital-based case-control study matched with age and sex.	2,395/9,580	Maternal smoking, alcohol, diabetes.
Berkowitz^2^	1995	America	Nested case-control study with 3 years follow-up	231 cases	Maternal smoking, BMI.
Damgaard^2^	2008	Denmark	Cohort study with 2 to 4 years follow-up.	128 cases	Maternal smoking, BMI.
Akre	1999	Sweden	Hospital-based case-control study matched with age and hospital birth.	2,782/13,916	Maternal smoking.
Beard	1984	America	Case-control study matched with year of delivery, maternal age, gravidity, parity.	81/159	Maternal smoking.
Preiksa	2005	Lithuania	Cohort study with 1 year follow-up.	69 cases	Maternal smoking.
Davies	1986	British	Hospital-based case-control study matched with age and hospital birth.	83/129	Maternal smoking, alcohol, diabetes.
Mcbirde	1991	British	Population-based case-control study matched with age and sex.	244/488	Maternal smoking, alcohol.
Carbone	2006	Italy	Population-based case-control study matched with age and city.	48/203	Maternal smoking, alcohol.
Wagner-Mahler	2011	France	Cohort study with 3 years follow-up.	76/137	Maternal smoking, alcohol drinking.
Shiono	1986	America	Cohort study with 3 years follow-up.	233/424	Maternal smoking.
Main^!^	2007, 2008	Denmark, Finland	Cohort study with 4 years follow-up.	95/185	Maternal smoking, alcohol, diabetes.

Jensen^1^ and Jensen^2^, Berkowitz^1^ and Berkowitz^2^, Damgaard^1^ and Damgaard^2^ are three pairs of studies, and each pair was published by the same author; some of the data are the same, but different risk factors were evaluated.

Main^!^ contains one article [48] and one response letter [49], and the data of alcohol is presented in [49].

### Maternal gestational smoking and the risk of cryptorchidism

**Fig 2:**
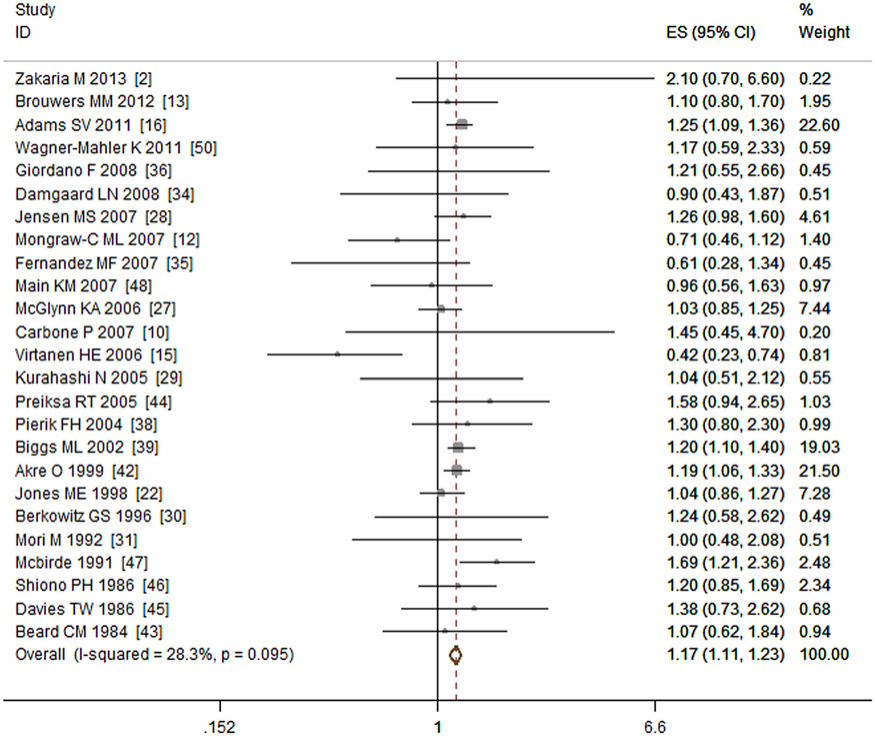
Forest plot for maternal gestational smoking and risk of cryptorchidism.

A total of 28 studies [2, 10, 12, 13, 15, 16, 22, 27–30, 31, 34–41, 42–48, 50] that included 11,900 cases investigated the relationship between maternal gestational smoking and the risk of cryptorchidism. Three studies [37, 40, 41] were excluded for duplicate data. Among the remaining 25 studies, nine were cohort studies, twelve were case—control studies and four were nested case—control studies. Our meta-analysis showed that maternal gestational smoking was associated with increased risk of cryptorchidism (OR = 1.17, 95% CI: 1.11–1.23; P < 0.01). That is, mothers who smoked during pregnancy had a 1.17 times higher odds of having a son with cryptorchidism than mothers who did not smoke. There was no obvious heterogeneity (I^2^ = 28.30%, P = 0.10) among studies. (See Fig. 2)

### Maternal gestational diabetes and risk of cryptorchidism

**Fig 3:**
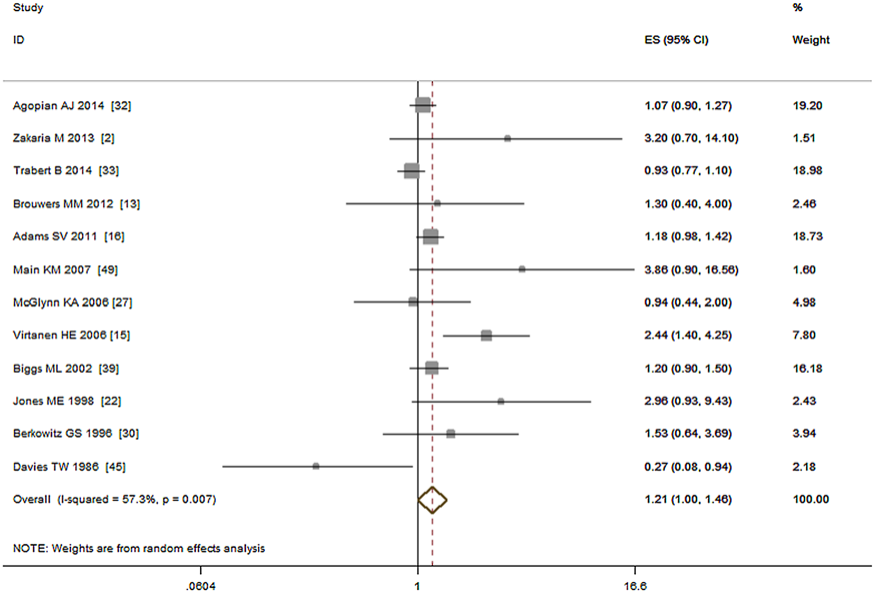
Forest plot for maternal gestational diabetes and risk of cryptorchidism.

Thirteen studies including 15,373 cases investigated the relationship between maternal gestational diabetes and risk of cryptorchidism [2, 13, 15, 16, 22, 27, 30, 32, 33, 39, 40, 45, 48]. Two studies, [30, 40] were published by same author in different years, we only used the data from [30]. Among the remaining 12 studies, five were cohort studies, five were case—control studies, and two were nested case—control studies. Our meta-analysis showed that gestational diabetes was marginal associated with increased risk of cryptorchidism (OR = 1.21, 95% CI: 1.00–1.46; P = 0.06). Moderate heterogeneity (I^2^ = 57.30%, P < 0.01) was seen among the studies. A Forest plot for maternal gestational diabetes and risk of cryptorchidism is shown in Fig. 3.

### Maternal gestational alcohol drinking and the risk of cryptorchidism

**Fig 4:**
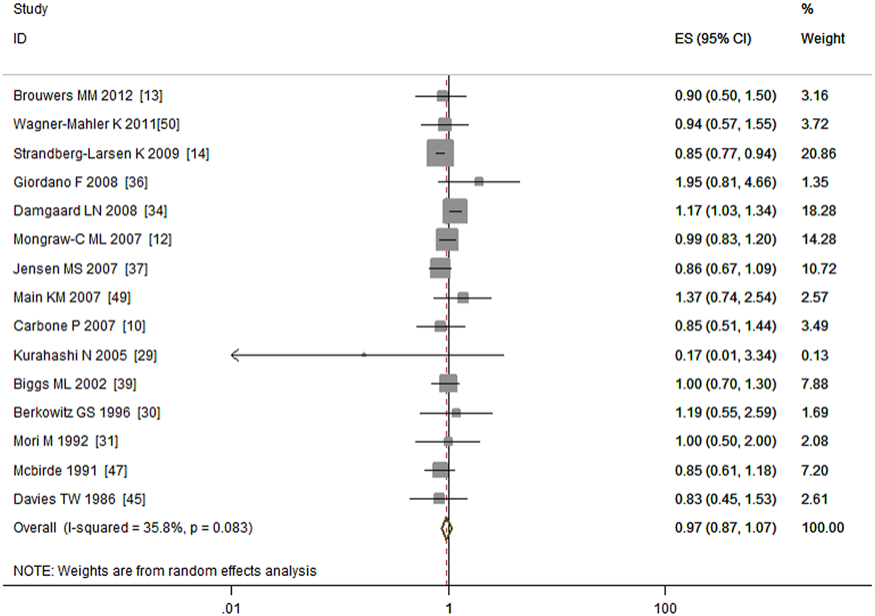
Forest plot for maternal gestational alcohol drinking and risk of cryptorchidism.

Fifteen studies investigated the association between maternal gestational alcohol drinking and the risk of cryptorchidism [10, 12–14, 29–31, 34, 36, 37, 39, 45, 47, 49, 50] including 5,601 cases in six cohort studies, eight case—control studies, and one nested case—control study. The meta-analysis did not find an association between maternal gestational drinking and the risk of cryptorchidism (OR = 0.97, 95% CI: 0.87–1.07; P = 0.54). Low to moderate heterogeneity (I^2^ = 35.80%, P = 0.08) was detected (Fig. 4).

### Maternal pre-pregnancy obesity and the risk of cryptorchidism

**Fig 5:**
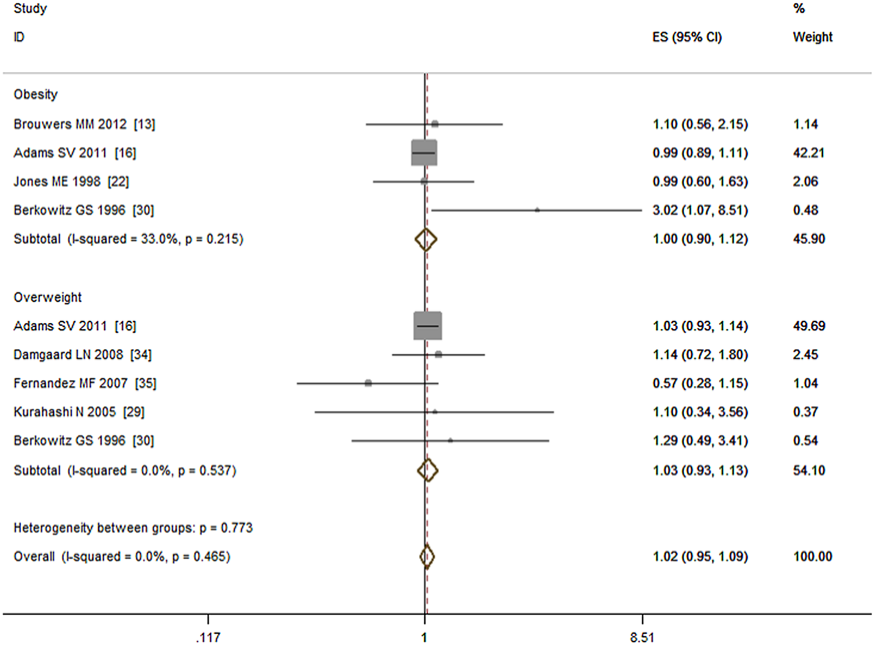
Forest plot for maternal pre-pregnancy BMI and risk of cryptorchidism. Subgroup analysis was conducted by dividing study participants into an obesity group (BMI >30 kg /m^2^) and an overweight group (25 kg /m^2^≤ BMI < 30 kg /m^2^).

Eight studies investigated the association between maternal pre-pregnancy BMI and the risk of cryptorchidism [13, 16, 22, 29, 30, 35, 40, 41]. A duplicate study [40] was removed from the analysis. The remaining seven reports included 4,397 cases comprising three case—control studies, three cohort studies, and one nested case—control study. Maternal pre-pregnancy BMI > 25 kg /m^2^ did not associated with increased cryptorchidism risk (OR = 1.02, 95% CI: 0.95–1.09; P = 0.67) compared with those who had a BMI < 25 kg /m^2^. No obvious heterogeneity (I^2^ = 0.00%, P = 0.47) was detected. Subgroup analyses showed that neither maternal obesity (BMI ≥ 30 kg /m^2^) nor pre-pregnancy overweight (25 kg /m^2^ ≤ BMI < 30 kg/m^2^) associated with the risk of cryptorchidism (OR = 1.00, 95% CI: 0.90–1.12; P = 0.94; I^2^ = 33.00% and OR = 1.03, 95% CI: 0.93–1.13; P = 0.61; I^2^ = 0.00%, respectively), see Fig. 5.

### Additional meta-analysis

**Fig 6:**
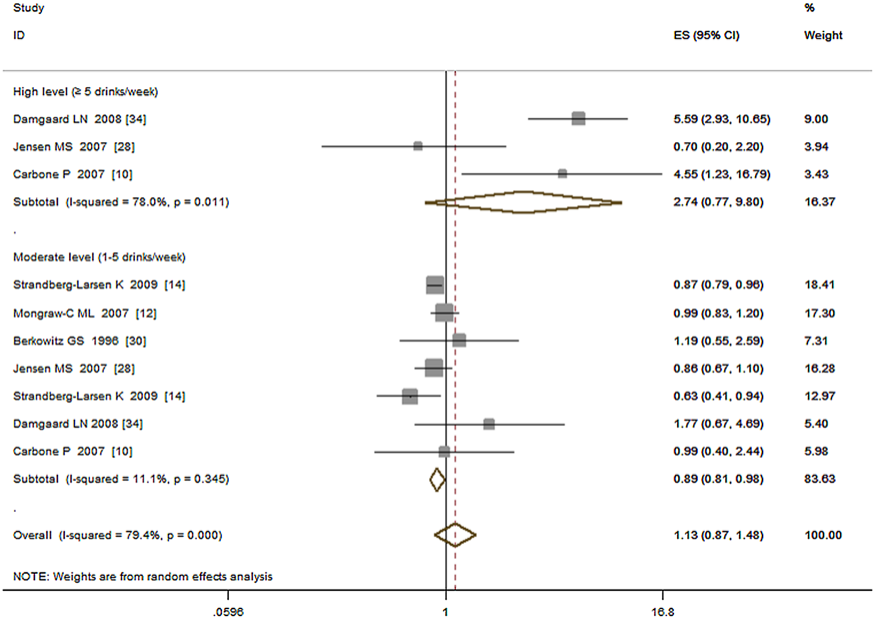
Forest plot for high and moderate vs. low exposure level of alcohol.

Several studies [12, 10, 14, 30, 34, 37] have investigated the risk of drinking alcohol and reported that high levels of alcohol consumption may increase the risk of cryptorchidism. We then pooled the ORs of high (≥ 5 drinks/week), moderate (1 to 5 drinks /week) vs. low (< 1 or no drinks /week) exposure levels separately and estimated the corresponding risk of cryptorchidism. The results showed that, high-level exposure was associated with increased risk of cryptorchidism (OR = 2.74, 95%CI: 0.77–9.80; P = 0.12; I^2^ = 78.00%) while moderate-level exposure associated decreased risk (OR = 0.89, 95%CI: 0.82–0.96; P < 0.01; I^2^ = 0.00%), see Fig. 6.

**Fig 7:**
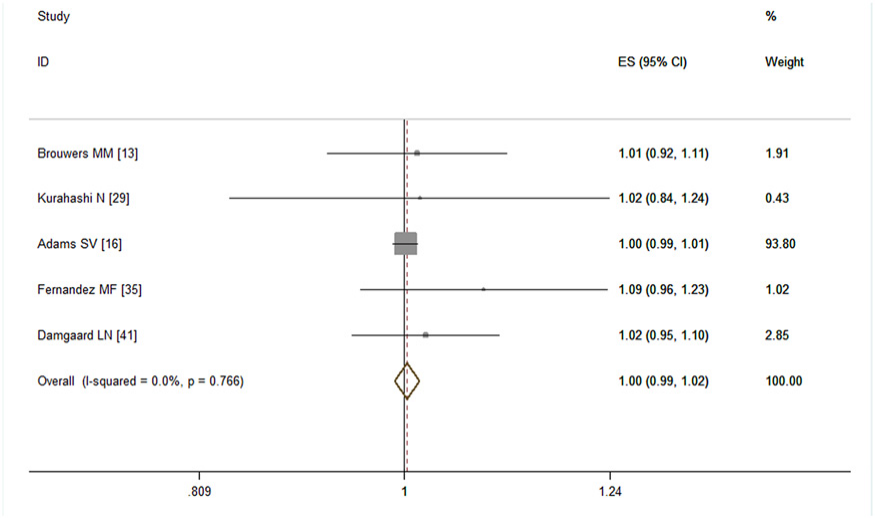
Forest plot for each 5 kg /m^2^ increase of BMI and risk of cryptorchidism.

Five studies [13, 16, 29, 40, 41] provided valid dose-response data for pre-pregnancy obesity. Our dose-response meta-analysis (Fig. 7) showed that every 5 kg /m^2^ increment of BMI was not associated with the risk of cryptorchidism (RR = 1.00, 95% CI: 0.99–1.02; P = 0.67). No obvious heterogeneity was detected (I^2^ = 0.00%, P = 0.77).

### Subgroup analysis

Because moderate heterogeneity was detected among the studies of alcohol drinking and diabetes, subgroup analyses on geographical region (European, Asian, America, Africa), study design (cohort, case—control, nested case-control), adjusted/crude ORs, and positive/negative results were done (Table 2). For alcohol drinking, high heterogeneity was found in European studies (I^2^ = 54.60%, P = 0.02), cohort studies (I^2^ = 69.80%, P < 0.01), studies adjusted for variables (I^2^ = 77.40%, P < 0.01), and studies reporting positive results (I^2^ = 93.10%, P < 0.01). For maternal diabetes, high heterogeneity was found in European studies (I^2^ = 67.50%, P = 0.02), studies adjusted for variables (I^2^ = 72.30%, P = 0.01), and studies reporting positive results (I^2^ = 90.20%, P < 0.01).

**Table 2:** The results of subgroup analysis of diabetes and drinking.

	Diabetes			Drinking		
Geographical region	I^2^	P-value^&^	ORs(95%CI)	I^2^	P-value^&^	ORs(95%CI)
European	67.50%	0.02	1.61(0.70, 3.67)	54.60%	0.02	0.96(0.83, 1.11)
America	0.00%	0.42	1.07(0.98, 1.18)	0.00%	0.90	1.00(0.86, 1.17)
Asian	–	–	–	26.00%	0.25	0.74(0.20, 2.73)
Africa	–	–	3.20(0.71, 14.36)	–	–	–
**Study design**						
Cohort	48.90%	0.10	1.07(0.85, 1.36)	69.80%	< 0.01	0.98(0.84, 1.14)
Case-control	66.40%	0.02	1.25(0.89, 1.76)	0.00%	0.68	0.93(0.78, 1.11)
Nested-CC^$^	0.00%	0.41	1.85(0.83, 3.93)	–	–	1.19(0.55, 2.58)
**Adjusted/crude ORs**						
Adjusted	72.30%	0.01	1.19(0.88, 1.60)	77.40%	< 0.01	0.98(0.80, 1.21)
Crude	45.30%	0.08	1.25(0.94, 1.26)	0.00%	0.91	0.97(0.85, 1.09)
**Positive/negative results**						
Positive	90.20%	< 0.01	0.87(0.1, 7.50)	93.10%	< 0.01	0.99(0.73, 1.36)
Negative	30.10%	0.17	1.12(0.98, 1.29)	0.00%	0.83	0.96(0.86, 1.06)

P-value^&^ is the P value of heterogeneity;

Nested-CC^$^ are nested case-control studies;

–: there is no or only one study in the group.

I^2^: the heterogeneity within subgroups, which varied from 0 to 100% and was described as low (0–40%), moderate (30–60%), substantial (50–90%), and considerable (75–100%) [23].

ORs: The odds ratio in each subgroup.

### Sensitivity analysis

Sensitivity analysis was conducted to evaluate the robustness of the results because some studies reported only RRs or HRs, and others had not adjusted for confounding variables. We found three articles [27, 33, 45], significantly influenced the diabetes results. Two of which were conducted in America and one in British. When one of them was omitted from the analysis the risk represented by diabetes was no longer significant. Another article [34], with an OR of 1.17 (95%CI: 1.03–1.34), influences the results of alcohol drinking obviously. When omitted it, the pooled ORs reached to statistical significance (OR = 0.90, 95%CI: 0.83–0.96). These results may be explained in part by ethnic or clinical diversity (i.e., the severity of diabetes). Sensitivity analysis did not result in any substantial change in the meta-analysis results on the influence of smoking and pre-pregnancy obesity.

### Publication bias

**Fig 8:**
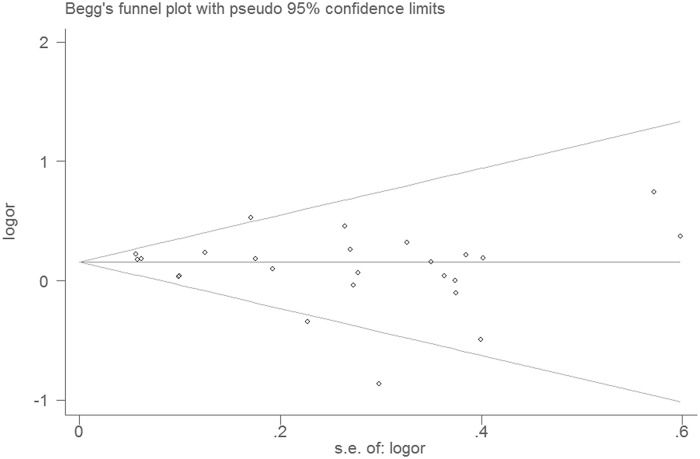
Begg’s test of studies reporting on maternal gestational smoking and risk of cryptorchidism.

Publication bias was evaluated by Egger’s test (P_1_) and Begg’s test (P_2_), which yielded no significant results for maternal gestational diabetes (P_1_ = 0.26, P_2_ = 0.47), smoking (P_1_ = 0.17, P_2_ = 0.30), drinking (P_1_ = 0.74, P_2_ = 0.51),or pre-pregnancy obesity (P_1_ = 0.52, P_2_ = 0.75). The results suggested no obvious publication bias in present meta-analysis. Fig. 8 shows the Begg’s test plot for studies that reported on maternal gestational smoking.

## Discussion

The present meta-analysis confirmed that maternal gestational smoking was associated with increased risk of cryptorchidism in newborn males. Sensitivity analysis supported the robustness of this result. This finding is consistent with two previous meta-analyses of maternal smoking and cryptorchidism. One [17] analyzed 13 studies by pooling the effect size but without adjusting any data or conducting additional analyses (e.g., heterogeneity testing) and without reporting fixed- or random-effect models. The second study [18], which was well designed but only included 18 studies, reported a summary OR (1.13, 95%CI: 1.02–1.25) similar to that seen here. Our updated meta-analysis, included 25 studies, had a rigorous design, and followed a widely accepted methodology, all of which support the validity of the results.

In this analysis, maternal gestational diabetes was linked with marginal increased risk of cryptorchidism, but the result should be interpreted with caution. Moderate heterogeneity was detected among the selected studies although a major source of heterogeneity was revealed by subgroup analysis. We also note that instability in sensitivity analysis can reduce the credibility of the results. After omitting one of the above three studies from the analysis, the effect of maternal gestational diabetes was no longer significant.

Alcohol drinking, overall, was not associated with increased risk of cryptorchidism, but high levels of alcohol drinking should be noted and discouraged. Previous studies [10, 34] reported an increased risk of cryptorchidism when mothers were heavy drinkers. The ORs of our additional meta-analysis including 6 of 15 studies showed that five or more drinks /week was associated with increased risk of cryptorchidism. But this result was statistically non-significant and was based on small numbers which needed further investigation. Moderate alcohol drinking may reduce the cryptorchidism risk. Our additional meta-analysis showed that 1 to 5 drinks /week was associated decreased risk of cryptorchidism. The unstable results of sensitivity analysis support this result. When omitted one article [34], the pooled ORs reached to statistic significance and showed reduced risk (OR = 0.90, 95%CI: 0.83–0.96) of cryptorchidism. These different effects by consumption level of alcohol on cryptorchidism were consistent with findings of study [51] of maternal alcohol drinking and risk of fetal growth and preterm birth. Further studies were needed to verify it.

The relationship between maternal pre-pregnancy obesity and the risk of cryptorchidism has been controversial. In our meta-analysis, we did not find evidence of such a relationship, and the sensitivity analysis indicates that our results are robust. We believe that the evidence of the studies evaluated here is not sufficient to confirm any relationship between pre-pregnancy obesity and the risk of cryptorchidism. In addition, no dose—response relationship was found between maternal pre-pregnancy BMI and the risk of cryptorchidism, which also supports this conclusion.

The descent of the testes normally occurs in two stages; a transabdominal stage at 7 to 15 weeks [52] and a transinguinal stage [6] at 20 to 35 weeks of pregnancy [52, 53]. Insulin-like peptide (INSL3), anti-Mullerian hormone (AMH), human chorionic gonadotropin (HCG), androgens and their receptor genes are all involved in these events [6, 53, 54, 55]. Any factors that can influence the levels of these hormones may affect descent of the testes. In our meta-analysis, maternal gestational smoking was found to be the only factor with clear evidence for significantly increasing the risk of cryptorchidism. Although the mechanism underlying this relationship is not known, previous studies may provide some clues. Previous studies [56, 57] found that maternal gestational smoking is associated with reduced levels of intact HCG and desert hedgehog (DHH) signaling in newborn males. HCG is needed for testicular descent and is used as a hormone treatment for cryptorchidism [6]. The effect of maternal gestational smoking on cryptorchidism risk may be mediated by reduction in HCG levels. Although the results for the effect of maternal diabetes were unstable, previous studies observed decreased serum sex hormone binding globulin (SHBG) levels [58–59] and increased insulin levels [60, 61] in umbilical cord blood in pregnant women with diabetes. Decreased SHBG levels may result in an increase of the ratio of non-SHBG-bound levels of estradiol and testosterone [62], which may then interfere with the descent of the testis. Whether down regulated expression of DHH or increased insulin levels in umbilical cord blood can cause cryptorchidism in newborn males is unclear. Further study is necessary.

### Strengths and limitations

Our meta-analysis followed a strict protocol, and the results provide an objective assessment of the relationships between maternal exposure to potential risk factors and cryptorchidism. Significant publication bias was not detected among the selected studies, adding to the strength of the results. Sensitivity analyses helped to avoid interference from confounding factors and to reduce reporting bias. Finally, additional meta-analyses (e.g., dose-response) allowed for investigation of possible relationships between the extent of exposure and the degree of risk.

This meta-analysis also has several limitations. Moderate heterogeneity was detected in studies of alcohol drinking and diabetes and sensitivity analysis revealed the result of alcohol drinking and diabetes was unstable. Although some variability is inevitable, this may influence the reliability of our results. In addition, at least half the studies did not adjust for confounding variables. Although we conducted subgroup meta-analyses to avoid bias, this may still affect our results. In addition, one [63] article did not find any relationships between maternal smoking and drinking with risk of cryptorchidism. Because this might have affected our results, we tried to contact the corresponding author for additional data, but with no response. Fourth, the Newcastle-Ottawa Scale [64] or other scales provided standard items for quality assessment; however, in our meta-analysis we included case-control, cohort, and nested case-control studies and did not assessed the quality of each study since there is not a standard specific to the assessment of nested case-control studies. Moreover, the studies we included usually contain multiple risk factors and some studies only adjusted confounders for the main risk factors (such as smoking) but not minor risk factors (such as diabetes). In this situation, the quality assessment tools are unusable.

### Conclusion

Smoking may be a risk factor for cryptorchidism; both parents should avoid fetal exposure to cigarette smoke. Maternal gestational diabetes may be (a) risk factor, but needs further research for confirmation. Drinking alcohol during pregnancy is not associated with increased risk of cryptorchidism, but five or more drinks a week may increase risk while one to five drinks a week may reduce the risk. No significant associations were found between maternal pre-pregnancy obesity or overweight and the risk of cryptorchidism.

## Supporting Information

S1 Dataset This table contains the whole data used in our meta-analysis. (XLSX)

S1 PRISMA Checklist (DOC)
